# Anorexia Caused by Hyperchloremic Metabolic Acidosis Following Ileal Conduit Diversion: A Case Report

**DOI:** 10.7759/cureus.74273

**Published:** 2024-11-22

**Authors:** Norihito Yoshida, Takanobu Sato, Shingo Ishii, Keisuke Yamazaki, Yasushi Ohashi

**Affiliations:** 1 Department of Nephrology, Toho University Sakura Medical Center, Sakura, JPN

**Keywords:** acute interstitial nephritis (ain), acute kidney injury (aki), acute tubular injury (ati), atypical anorexia, chronic kidney disease (ckd), hyperchloremic metabolic acidosis, ileal conduit, intermittent hemodialysis (ihd), vancomycin-induced nephrotoxicity (vin)

## Abstract

Hyperchloremic metabolic acidosis is a known complication following ileal conduit urinary diversion, often arising from urinary reabsorption in the ileum, which leads to chloride retention and bicarbonate loss and, though often asymptomatic, can produce clinically significant symptoms, particularly in patients with underlying renal impairment. A 75-year-old woman with a history of bladder cancer underwent cystectomy with ileal conduit diversion and presented on postoperative day 47 with anorexia, hypotension, and weight loss; laboratory findings revealed hyperchloremic metabolic acidosis with elevated serum chloride. The patient's acidosis gradually improved with sodium bicarbonate and Ringer's solution, stabilizing her blood pressure, creatinine, and acid-base balance, and she was discharged with outpatient follow-up. This case highlights the role of urinary reabsorption in the ileal conduit as a cause of bicarbonate loss and acidosis exacerbation, particularly in patients with renal impairment, while the sodium-chloride gap, although supplementary, provided additional insights into acidosis progression and facilitated early detection in this case. Hyperchloremic metabolic acidosis following ileal conduit diversion warrants vigilant monitoring and timely intervention, with bicarbonate supplementation playing a central role in treatment to optimize clinical outcomes in patients with compromised renal function.

## Introduction

The ileal conduit is a widely used option for urinary diversion following radical cystectomy for bladder cancer, providing anatomical and functional reconstruction without the need for a stoma bag or ureteral appliance [1,2]. Hyperchloremic metabolic acidosis is believed to occur due to urine retention within the ileum, where ion reabsorption happens through the intestinal mucosa [3]. The first case of this complication was reported in 1931, and subsequent studies have documented an incidence rate of 14.8% within the first month postoperatively, with potential improvement over the course of one year owing to mucosal adaptation and metabolic compensatory mechanisms [4,5]. Although hyperchloremic metabolic acidosis associated with the ileal conduit is clinically asymptomatic in most cases, about 10% of patients may develop clinically significant symptoms [6]. This condition can occur even in patients with normal renal function, but the risk is substantially higher in those with impaired renal function [7,8].

## Case presentation

A 75-year-old female patient who underwent cystectomy with ileal conduit diversion for bladder cancer presented to our internal medicine outpatient clinic on postoperative day 47. Two weeks before this presentation, she began to experience anorexia. One week before her visit, she attended a routine check-up at her primary care clinic, where hypotension was noted, leading to a reduction in her antihypertensive medication. Despite this adjustment, her symptoms persisted, and she experienced a 2 kg weight loss from her postoperative baseline, prompting further evaluation. Her past medical history included chronic kidney disease (CKD), hypertension, hypothyroidism, and a history of left breast cancer surgery. CKD was classified as stage 3b, likely secondary to benign nephrosclerosis, given her long-standing history of hypertension. She was independent in activities of daily living (ADL), with no history of smoking or alcohol use, and reported no known allergies. On physical examination, the patient's height was 146 cm, and her weight was 40.6 kg. Vital signs revealed a temperature of 35.8°C, a blood pressure of 101/67 mm Hg, a pulse rate of 113 beats per minute, a respiratory rate of 28 breaths per minute, and an oxygen saturation of 99% while breathing ambient air. She was alert, with pallor of the palpebral conjunctiva but no scleral icterus. The thyroid was normal in size and non-tender, with no cervical tenderness or dysphagia. There was no axillary lymphadenopathy. Lung auscultation revealed clear breath sounds bilaterally, and heart sounds were regular without murmurs. The abdomen was flat and soft, with no tenderness or distention. There was no edema in the lower extremities. Venous blood gas analysis revealed a pH of 6.983, a bicarbonate level of 5.7 mmol/L, a base excess of -24.5 mmol/L, and an anion gap of 11.3 mmol/L, consistent with non-anion gap metabolic acidosis. Serum chloride was elevated at 117 mEq/L, while blood urea nitrogen (BUN) and serum creatinine were elevated at 102 mg/dL and 2.56 mg/dL, respectively, with an estimated glomerular filtration rate (eGFR) of 15 mL/min/1.73 m², indicating acute kidney injury. The fractional excretion of sodium (FeNa) was 1.2%, with no findings suggestive of pre-renal etiology. Thyroid function tests were within normal limits, and there were no indications of adrenal insufficiency, with the adrenocorticotropic hormone (ACTH) at 9.1 pg/mL and cortisol at 16.1 µg/dL. Screening for anti-neutrophil cytoplasmic antibody (ANCA)-associated vasculitis and multiple myeloma was also negative, with negative results for Bence-Jones protein, antinuclear antibody (ANA), anti-glomerular basement membrane antibody (anti-GBM Ab), myeloperoxidase anti-neutrophil cytoplasmic antibody (MPO-ANCA), and proteinase 3 anti-neutrophil cytoplasmic antibody (PR3-ANCA). The urinary anion gap was positive (Table 1).

**Table 1:** Laboratory findings WBC: white blood cell; Hb: hemoglobin; Plt: platelet; CRP: C-reactive protein; TP: total protein; Alb: albumin; AST: aspartate aminotransferase; ALT: alanine aminotransferase; ALP: alkaline phosphatase; LDH: lactate dehydrogenase; γ-GTP: gamma-glutamyl transferase; Na: sodium; K: potassium; Cl: chloride; BUN: blood urea nitrogen; Cr: creatinine; eGFR: estimated glomerular filtration rate; Ca: calcium; P: phosphorus; BG: blood glucose; IgG: immunoglobulin G; IgA: immunoglobulin A; IgM: immunoglobulin M; ACTH: adrenocorticotropic hormone; TSH: thyroid-stimulating hormone; FT3: free triiodothyronine; FT4: free thyroxine; BJP: Bence-Jones protein; ANA: antinuclear antibody; anti-GBM Ab: anti-glomerular basement membrane antibody; MPO-ANCA: myeloperoxidase anti-neutrophil cytoplasmic antibody; PR3-ANCA: proteinase 3 anti-neutrophil cytoplasmic antibody; HCO₃⁻: bicarbonate; BE: base excess; AG: anion gap; WBC (urine): white blood cell in urine; RBC (urine): red blood cell in urine; urine Na: urine sodium; urine K: urine potassium; urine Cl: urine chloride; urine Cr: urine creatinine; HPF: high-power field

Test	Result	Normal range
WBC	10,670/μL	3,500-9,000/μL
Hb	10.5 g/dL	13.0-17.0 g/dL (male)/12.0-15.0 g/dL (female)
Plt	238,000/μL	150,000-350,000/μL
CRP	0.11 mg/dL	<0.3 mg/dL
TP	7.9 g/dL	6.5-8.0 g/dL
Alb	4.2 g/dL	3.8-5.3 g/dL
AST	13 IU/L	10-40 IU/L
ALT	8 IU/L	5-40 IU/L
ALP	75 U/L	30-120 U/L
LDH	149 U/L	120-240 U/L
γ-GTP	29 IU/L	<50 IU/L (male)/<32 IU/L (female)
Na	134 mEq/L	135-145 mEq/L
K	4.9 mEq/L	3.5-5.0 mEq/L
Cl	117 mEq/L	98-108 mEq/L
BUN	102 mg/dL	8-20 mg/dL
Cr	2.56 mg/dL	0.65-1.07 mg/dL (male)/0.46-0.79 mg/dL (female)
eGFR	15 mL/min/1.73 m²	>60 mL/min/1.73 m²
Ca	10.4 mg/dL	8.8-10.2 mg/dL
P	5.4 mg/dL	2.5-4.5 mg/dL
BG	116 mg/dL	70-110 mg/dL (fasting)
IgG	1756 mg/dL	870-1700 mg/dL
IgA	207 mg/dL	110-410 mg/dL
IgM	54 mg/dL	35-220 mg/dL
ACTH	9.1 pg/mL	7.2-63.3 pg/mL
Cortisol	16.1 µg/dL	6.2-19.4 µg/dL (morning)
TSH	0.29 µIU/mL	0.5-5.0 µIU/mL
FT3	2.18 pg/mL	2.0-4.0 pg/mL
FT4	1.33 ng/dL	0.9-1.7 ng/dL
BJP	Negative	Negative
ANA	40	<40 (negative)
Anti-GBM Ab	<1.5 U/mL	<3.0 U/mL
MPO-ANCA	<0.2 IU/mL	<3.5 IU/mL
PR3-ANCA	<0.6 IU/mL	<3.5 IU/mL
Venous blood gas analysis		
pH	6.983	7.35-7.45
HCO₃⁻	5.7 mmol/L	22-26 mmol/L
BE	-24.5 mmol/L	-2 to +2 mmol/L
Lactate	1.12 mmol/L	0.5-2.0 mmol/L
AG	11.3 mmol/L	8-16 mmol/L
Urine analysis		
pH	6.5	4.6-8.0
Specific gravity	1.011	1.005-1.030
WBC (urine)	5-9/HPF	0-4/HPF
RBC (urine)	1-4/HPF	0-4/HPF
Urine Na	61 mEq/L	N/A
Urine K	24.8 mEq/L	N/A
Urine Cl	15 mEq/L	N/A
Urine Cr	126.17 mg/L	N/A
Hyaline casts	+	Negative

No tenderness was elicited upon the spine or costovertebral angle percussion. Electrocardiography (ECG) demonstrated sinus rhythm at 106 bpm with no ST-segment changes. The chest X-ray was unremarkable. Renal ultrasonography revealed bilateral renal atrophy without hydronephrosis. Renal artery stenosis was not observed, and renal vein Doppler ultrasound showed continuous waveforms. Echocardiography demonstrated an ejection fraction of 50%, mild aortic regurgitation, and mild tricuspid regurgitation, with no pericardial effusion. Non-contrast CT imaging of the chest, abdomen, and pelvis was performed. The bladder was absent, and an ileal conduit was observed in the right lower abdomen. No lymphadenopathy or findings suspicious for malignancy were identified. Additionally, there were no abnormalities in the lung fields, gallbladder, or bile ducts, and there were no signs of bowel obstruction. Calcifications were identified in the aorta and coronary arteries.

**Figure 1:**
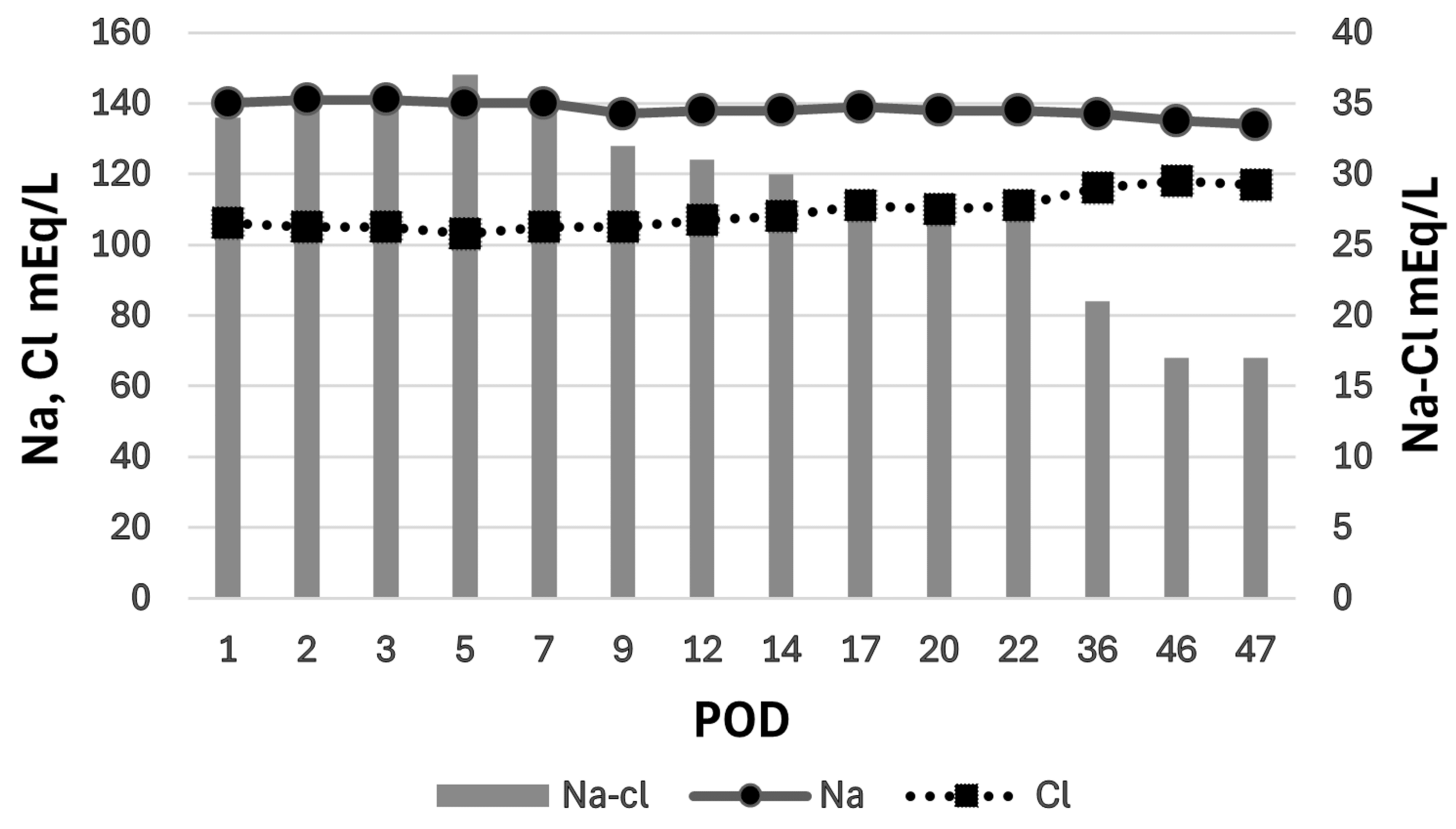
Postoperative to pre-admission changes in serum Na, Cl, and Na-Cl gap The figure shows the postoperative trends in serum Na, Cl, and the Na-Cl gap, tracked from surgery to pre-admission. Serum Na levels remained relatively stable throughout the postoperative period, while Cl levels showed a gradual increase. The Na-Cl gap, indicated by the gray bars, progressively narrowed, eventually falling below 36. This narrowing of the Na-Cl gap aligns with the patient's developing metabolic acidosis and associated symptoms, prompting hospital admission on postoperative day 47 Na: sodium; Cl: chloride

Prior to admission, the patient had been hospitalized for postoperative monitoring and training in urinary management during the first three weeks following surgery. During this period, her sodium-chloride (Na-Cl) gap remained stable with no significant fluctuations. However, approximately three weeks postoperatively, the Na-Cl gap gradually decreased, eventually falling below 36, indicating a trend toward metabolic acidosis. Concurrently, she began experiencing anorexia, prompting a referral to our facility on postoperative day 47, where she was admitted the same day for further management (Figure 1).

**Figure 2:**
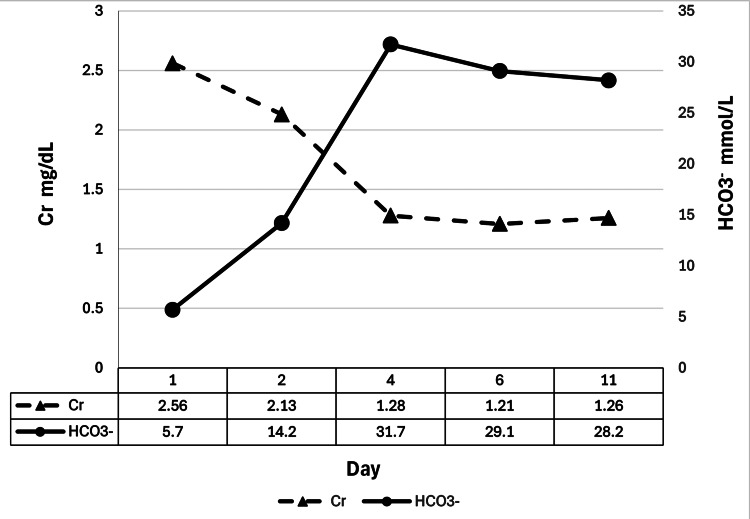
Post-admission course of serum Cr and HCO₃⁻ levels The figure illustrates the changes in serum Cr and HCO₃⁻ levels following hospital admission. Serum Cr levels (dashed line) gradually declined post-admission, indicating an improvement in renal function, while serum HCO₃⁻ levels (solid line) steadily increased, signifying effective correction of metabolic acidosis. These trends underscore the therapeutic efficacy of sodium HCO₃⁻ administration, with the stabilization of both Cr and HCO₃⁻ levels observed by day 11 Cr: creatinine; HCO₃⁻: bicarbonate

Given her hyperchloremic metabolic acidosis, likely resulting from urinary reabsorption in the ileal conduit, along with anorexia and hypotension, treatment was initiated with sodium bicarbonate and Ringer's solution. An 8.4% sodium bicarbonate solution was administered at 250 mL/day. By the second hospital day, her blood pressure showed an upward trend, and serum creatinine levels began improving; however, correction of the acidosis remained incomplete, necessitating continued bicarbonate infusion. On the third hospital day, she resumed oral intake. Sodium bicarbonate was then transitioned to an oral maintenance dose of 1000 mg/day on the fourth hospital day. By the sixth day of hospitalization, stabilization was achieved in her serum creatinine and bicarbonate levels, along with her oral intake. She was discharged on the 12th hospital day with outpatient follow-up, including continued oral sodium bicarbonate supplementation at 1000 mg/day (Figure 2).

## Discussion

In this case, no decline in the Na-Cl gap was observed immediately postoperatively or during the initial hospitalization. Preoperative blood tests indicated CKD, but there was no evidence of metabolic acidosis, as no significant narrowing of the Na-Cl gap was observed. This suggests that the patient's renal function, while impaired, retained sufficient acid-excretion capacity before surgery. However, approximately one month post-surgery, a gradual decrease in the Na-Cl gap was noted, coinciding with the development of metabolic acidosis. The timing of acidosis exacerbation aligned with the onset of anorexia, and a downward trend in blood pressure was also observed, suggesting a possible impact of acidosis. Following admission, the patient's symptoms improved with alkalinization therapy, supporting the hypothesis that her symptoms were primarily attributable to acidosis.

This hyperchloremic metabolic acidosis can be explained by urinary reabsorption through the ileal conduit, leading to bicarbonate loss. In cases of urine reabsorption, the chloride/bicarbonate (Cl^-^/HCO_3_^-^) exchange channel (SLC26A3) located in the intestinal epithelium is activated, promoting Cl^-^ reabsorption and HCO_3_^-^ secretion, resulting in a net loss of bicarbonate and the development of metabolic acidosis. Additionally, in patients with renal impairment, compromised excretion of NH_3_^-^ and H^+^ in the proximal tubules may further exacerbate acidosis [3,9,10].

In this patient, extensive calcifications associated with nephrosclerosis were detected on preoperative CT, indicative of CKD and suggesting reduced H+ excretion. The main differential diagnoses for hyperchloremic metabolic acidosis include (1) impaired renal H^+^ excretion or NH_3_^-^ production, (2) HCO_3_^-^ loss from the kidneys or gastrointestinal tract, (3) anion loss in urine, and (4) excessive Cl_- _load [11,12]. However, the patient had no history of diarrhea, use of medications associated with HCO₃⁻ loss, or saline infusion, and the absence of low potassium and relevant medications made renal tubular acidosis unlikely. The positive urinary anion gap observed in this case suggests a reduction in acid excretion due to tubular dysfunction associated with acute kidney injury, rather than chronic renal tubular acidosis, as evidenced by the absence of hypokalemia. Therefore, the primary mechanism of acidosis in this case was attributed to HCO₃⁻ loss mediated by the Cl⁻/HCO₃⁻ exchange in the ileum.

One unique strength of this case report is the detailed tracking of the Na-Cl gap from the postoperative period through the initial clinical consultation, allowing for a comprehensive understanding of the patient's metabolic changes over time. Postoperatively, the Na-Cl gap showed no significant changes in the first week, but a progressive narrowing was observed by the first month, closely correlating with the emergence of symptoms. This pattern highlights the importance of monitoring the Na-Cl gap as a marker of impending metabolic acidosis, as prompt recognition allowed for timely intervention in this case.

As a valuable marker of acid-base balance, the Na-Cl gap provides quick insights into potential acidosis when values fall below 36 or alkalosis when they rise above 36. In clinical practice, its use can facilitate the early recognition of metabolic disturbances; in this case, the Na-Cl gap narrowing observed during the initial consultation prompted further blood gas analysis, which confirmed the presence of hyperchloremic metabolic acidosis [13].

Treatment included sodium bicarbonate supplementation to correct the acidosis, with plans for long-term follow-up [10]. Although lifelong supplementation may be necessary, titrating to the lowest effective maintenance dose will be essential. In some cases, bicarbonate supplementation may eventually be discontinued as mucosal adaptation and compensatory mechanisms can lead to an improvement in acidosis prevalence over time, with a potential decrease in incidence after one year [5,14]. In patients with urinary tract malignancies, concurrent CKD is common, making compensation for metabolic changes more challenging and increasing the risk of worsening metabolic acidosis, as seen in this case. Therefore, narrowing of the Na-Cl gap should prompt immediate blood gas analysis to ensure early diagnosis and management of acidosis.

## Conclusions

This case illustrates hyperchloremic metabolic acidosis as a serious complication following ileal conduit urinary diversion, especially in patients with CKD. In CKD, reduced acid excretion due to impaired ammoniagenesis leads to acid retention and bicarbonate depletion. This is further exacerbated by chloride reabsorption in the ileum, which increases bicarbonate loss and contributes to worsening acidosis. Monitoring the Na-Cl gap proved effective for early acidosis detection, with its narrowing closely correlating with symptom onset. Sodium bicarbonate supplementation successfully managed acidosis, and long-term care may benefit from customized dosing and potential tapering as mucosal adaptation occurs. This case emphasizes the need for vigilance in managing metabolic complications in urinary diversion patients, particularly those with CKD, to improve outcomes and prevent related morbidity.
